# Microbial Life in the Deep Subsurface Aquifer Illuminated by Metagenomics

**DOI:** 10.3389/fmicb.2020.572252

**Published:** 2020-09-03

**Authors:** Vitaly V. Kadnikov, Andrey V. Mardanov, Alexey V. Beletsky, Olga V. Karnachuk, Nikolai V. Ravin

**Affiliations:** ^1^Institute of Bioengineering, Research Center of Biotechnology of the Russian Academy of Sciences, Moscow, Russia; ^2^Laboratory of Biochemistry and Molecular Biology, Tomsk State University, Tomsk, Russia

**Keywords:** deep subsurface, metagenome, uncultivable bacteria, candidate phylum, microbial diversity

## Abstract

To get insights into microbial diversity and biogeochemical processes in the terrestrial deep subsurface aquifer, we sequenced the metagenome of artesian water collected at a 2.8 km deep oil exploration borehole 5P in Western Siberia, Russia. We obtained 71 metagenome-assembled genomes (MAGs), altogether comprising 93% of the metagenome. Methanogenic archaea accounted for about 20% of the community and mostly belonged to hydrogenotrophic *Methanobacteriaceae;* acetoclastic and methylotrophic lineages were less abundant. ANME archaea were not found. The most numerous bacteria were the *Firmicutes*, *Ignavibacteriae*, *Deltaproteobacteria*, *Chloroflexi*, and *Armatimonadetes*. Most of the community was composed of anaerobic heterotrophs. Only six MAGs belonged to sulfate reducers. These MAGs accounted for 5% of the metagenome and were assigned to the *Firmicutes*, *Deltaproteobacteria*, *Candidatus* Kapabacteria, and *Nitrospirae*. Organotrophic bacteria carrying cytochrome *c* oxidase genes and presumably capable of aerobic respiration mostly belonged to the *Chloroflexi*, *Ignavibacteriae*, and *Armatimonadetes.* They accounted for 13% of the community. The first complete closed genomes were obtained for members of the *Ignavibacteriae* SJA-28 lineage and the candidate phylum Kapabacteria. Metabolic reconstruction of the SJA-28 bacterium, designated *Candidatus* Tepidiaquacella proteinivora, predicted that it is an anaerobe growing on proteinaceous substrates by fermentation or anaerobic respiration. The *Ca*. Kapabacteria genome contained both the sulfate reduction pathway and cytochrome *c* oxidase. Presumably, the availability of buried organic matter of Mesozoic marine sediments, long-term recharge of the aquifer with meteoric waters and its spatial heterogeneity provided the conditions for the development of microbial communities, taxonomically and functionally more diverse than those found in oligotrophic underground ecosystems.

## Introduction

Deep subsurface environments provide the largest habitats for prokaryotes in terms of size (Whitman et al., 1998). According to recent estimates (Magnabosco et al., 2018), the deep subsurface biosphere contains from 12 to 20% of the total biomass of microorganisms on Earth, mostly in the continental subsurface. Both the deep sub-seafloor and terrestrial subsurface habitats contain a variety of functionally active microbial communities, the existence of which is limited only by temperature increase with depth and is possible at depths of up to 3–5 km (Ghiorse and Wilson, 1988; Parkes et al., 2000; Takai et al., 2001; Teske, 2005; Itävaara et al., 2011; Lomstein et al., 2012; Bomberg et al., 2015). Such microbial communities may be independent of the supply of organic matter from the surface and exist autonomously for hundreds of millions of years (Chivian et al., 2008; Edwards et al., 2012). Depending on the type of rocks, underground microbial communities may be lithoautotrophic or organotrophic (Fredrickson and Hicks, 1987). In the lithoautotrophic communities characteristic of igneous rocks, the main source of energy is molecular hydrogen of abiotic origin, and the content of organic carbon is extremely low. Such microbial communities are usually characterized by low diversity and contain sulfate-reducing or methanogenic microorganisms (Moser et al., 2005; Chivian et al., 2008). In sedimentary rocks, organic matter buried since their formation provides energy sources and organic carbon, which enable the development of various organotrophic microorganisms. The best-studied example of such ecosystems is the formation waters of oil reservoirs, which harbor diverse communities of bacteria and archaea (Orphan et al., 2003; Lewin et al., 2014; Hu et al., 2016). The microbial communities of deep terrestrial subsurface ecosystems have been investigated in a number of studies (Miettinen et al., 2015; Hubalek et al., 2016; Magnabosco et al., 2016; Wu et al., 2016; Colman et al., 2017; Momper et al., 2017). Deep subsurface ecosystems can be accessed through drilled boreholes penetrating deep strata. Particularly, oil-exploration boreholes, drilled to a depth of several kilometers, provide a unique opportunity to access microbial communities of deep subsurface aquifers. The Western Siberian megabasin, formed from marine sediments of the Mesozoic era, is one of the largest oil and gas reserves in the world (Vyssotski et al., 2006). This region also harbors huge underground waterbodies located at depths of 1–3 km (Novikov and Sukhorukova, 2015). A number of oil-exploration boreholes have been drilled here since the 1950s. Some of them remain open and are currently used as sources of artesian thermal waters, thus providing a unique opportunity for sampling microbial communities of subsurface aquifers. Three such deep subsurface aquifers have been studied by molecular and cultivation approaches. The microbial community of an aquifer broached by the 3P borehole in the Parabel district (Tomsk region, Russia) was characterized by 16S rRNA profiling and cultivation; it was found to consist mostly of chemolithoautotrophs, – sulfate-reducing *Firmicutes* and hydrogenotrophic methanogenic archaea (Frank et al., 2016). The microbial community of the second aquifer, accessed via the 1-R borehole in the Byelii Yar district, has been analyzed by metagenomics. This community was more complex: it lacked methanogens and comprised sulfate-reducing *Firmicutes* and *Deltaproteobacteria*, as well as various presumably organotrophic members of the phyla *Chlorofexi*, *Ignavibacteriae*, *Ca.* Aminicenantes, *Ca.* Riflebacteria and BRC1 (Kadnikov et al., 2018). The metagenomic data enabled genome-based characterization of members of *Ca.* Aminicenantes, *Ca.* Riflebacteria and BRC1 (Kadnikov et al., 2018, 2019a,b) and cultivation of an enigmatic uncultured firmicute, *Ca.* Desulforudis audaxviator (Karnachuk et al., 2019), previously known by its genome retrieved from a deep gold mine in South Africa, where it formed a single-species hydrogen-driven ecosystem (Chivian et al., 2008).

The third site, borehole 5P in the Chazhemto district, was analyzed by 16S rRNA profiling (Kadnikov et al., 2017a,b). It harbored a rather different microbial community, consisting of various lineages of the phyla *Firmicutes, Ignavibacteria, Chloroflexi, Bacteroidetes*, and *Proteobacteria*, phylogenetically distant from cultured species, and methanogenic archaea of the genera *Methanothermobacter* and *Methanosaeta*. Known sulfate reducers were not identified. However, the lack of metagenomics data prevents us from predicting the functional roles of the majority of this community represented by uncultured lineages and obtaining insights into the ecology of this subsurface aquifer.

Here we report a metagenomics analysis of the deep subsurface aquifer accessed via the 5P borehole. We successfully recovered high-quality metagenome-assembled genomes of the vast majority of community members that enabled us to perform an accurate metabolic reconstruction and propose an ecological model of the deep subsurface aquifer.

## Materials and Methods

### Site Description

The oil-exploration borehole 5P is located near the village of Chazhemto in the Tomsk region of Russia (58.077481N, 82.836091E). The borehole was drilled in the 1950s to a depth of 2.8 km. It penetrated Quaternary sediments near the surface followed by Paleogene, Cretaceous and Jurassic sediments to a depth of ∼2.5 km. Upon passing Jurassic sedimentary rocks, the borehole entered the Palaeozoic basement (Banks et al., 2014). According to oil exploration practices of the 1950s, the borehole was tested by a bottom-up casing perforation method. This method involves perforating the walls of the pipe and testing for the outflow. After testing, the section was sealed with cement, and then the next section was tested in the same way. The last tested section is assumed to be located at a depth of more than 2 km. Therefore, the water is expected to originate from an aquifer system located at a depth of 2–2.5 km in the Mesozoic sedimentary rocks. The artesian water flows out spontaneously under natural pressure with the rate of about 11.5 m^3^ per day and is used by local populations as a source of mineral water through a closed wellhead with a set of connectors.

### Sampling, Field Measurements, Chemical Analyses, and DNA Isolation

Water samples were collected at the wellhead on April 30, 2016. Water temperature, pH and Eh were determined on site using an HI 8314 pH meter (Hanna Instruments, Germany). For chemical analysis, the water samples were filtered through a 0.2-μm sterile filter (Merck Millipore, Germany) and analyzed by ICP-MS and ion chromatography as reported previously (Kadnikov et al., 2017a). Gas content was determined using a Kristall-5000.1 gas chromatograph (Russia) equipped with a katharometer detector. Microbial biomass from 25 L of groundwater was collected by filtration on 0.22 μm cellulose nitrate membranes (Sartorius, Germany) using a Sartorius filtration unit. The filters were frozen in liquid nitrogen and then ground and melted with TE buffer in a water bath at 37°C. Total DNA was extracted using a Power Soil DNA isolation kit (MO BIO Laboratories, Inc., Carlsbad, CA, United States). About 1 μg of DNA was isolated.

### Sequencing of Metagenomic DNA Using the Illumina Platform and Assembly of MAGs

Sequencing of a paired-end (2 × 250 bp) TruSeq DNA library using an Illumina HiSeq2500 platform generated 57,579,354 read pairs (Kadnikov et al., 2017c). Adapters were trimmed using Cutadapt v.1.14 (Martin, 2011). Sequencing reads were preprocessed by trimming low-quality sequences (Q < 33) using Sickle version 1.33^1^. Trimmed reads were merged with FLASH v.1.2.11 (Magoć and Salzberg, 2011). Resulting merged and unmerged reads (about 16.9 Gbp in total) were *de novo* assembled using metaSPAdes version 3.7.1 (Bankevich et al., 2012) into 185,598 contigs longer than 500 bp.

Contigs longer than 2000 bp were binned into MAGs using the program CONCOCT v.0.4.1 (Alneberg et al., 2014). The completeness of obtained MAGs and their possible contamination were estimated using CheckM v.1.05 (Parks et al., 2015) with lineage-specific marker genes. The assembled MAGs were taxonomically assigned using the GTDB-Tk version 0.1.3 tool and Genome Taxonomy database (GTDB) (Parks et al., 2018).

The 16S rRNA genes in the contigs were identified using CheckM. The search for continuations of contigs representing fragments of 16S rRNA genes using Bandage v.0.8.0 program (Wick et al., 2015) allowed linkage of 16S rRNA genes to most of the genome bins where 16S rRNA carrying contigs were missing.

### Nanopore Sequencing and Improved Assembly of MAGs

Metagenomic DNA was additionally sequenced on a MinION system (Oxford Nanopore, United Kingdom) using the 1D Genomic DNA by ligation protocol. Sequencing generated 1,418,419 reads of a total length of about 1.54 Gbp. These long reads were used to assemble the Illumina contigs of a given MAG into longer sequences. For this purpose, the MinION reads exhibiting high sequence similarity to the contigs of a given MAG were selected using BWA v.0.7.15 (Li and Durbin, 2010). Then the contigs were merged using the npScarf program (Cao et al., 2017). The consensus sequence was polished using Pilon v.1.22 (Walker et al., 2014) with mapping of Illumina reads back to the assembled sequence using Bowtie 2 (Langmead and Salzberg, 2012).

### Annotation and Analysis of MAGs

Gene search and annotation of MAGs was performed using the RAST server 2.0 (Brettin et al., 2015), followed by manual correction of the annotation by the comparison of predicted protein sequences with the National Center for Biotechnology Information (NCBI) databases. Signal peptides were predicted by Signal P v.4.1^2^ and PRED-TAT^3^, and the presence of transmembrane helices was predicted by TMHMM v. 2.0^4^.

### Genome-to-Genome Distance Estimation and Phylogenetic Analysis

The average amino acid identity (AAI) between the selected genomes was calculated using the aai.rb script from the Enveomics Collection (Rodriguez-R and Konstantinidis, 2016).

The GTDB-Tk v.0.1.3 tool was used to find single-copy marker genes in the assembled MAGs and to construct a multiple alignment of concatenated single-copy gene sequences, comprising those from a given MAG and all species from the GTDB. In some cases, genomes of related organisms missing in GTDB were additionally included in this analysis. A selected part of the multiple alignment built into GTDB-Tk was used to construct a phylogenetic tree in PhyML v. 3.3 (Guindon et al., 2010) with default parameters. The level of support for internal branches was assessed using the Bayesian test in PhyML.

### Nucleotide Sequence Accession Numbers

The metagenomic sequences obtained in this study have been deposited in the NCBI Sequence Read Archive under the accession numbers SRR6186653 (Illumina reads) and SRR11854357 (MinION reads). The annotated sequences of high-quality MAGs have been deposited in the GenBank database under the BioProject PRJNA414521.

## Results and Discussion

### Water Chemistry

The physical and chemical characteristics of the water collected at the 5P borehole in April 2016 are shown in Table 1. The water temperature was surprisingly low (19.8°C), considering a typical thermal gradient of 20–30°C per km and an expected 2 km depth of the productive horizon. However, the water may cool when passing through the borehole, and it should be noted that over the past 15 years of observation, the water temperature has decreased by about 10°C, and the flow rate up the well has also decreased, which may be due to clogging of the well pipe. The water had a near neutral pH (about 7.5) and was highly reduced (Eh – 329 mV). The ionic content of the water was dominated by sodium and chloride, with subsidiary calcium. The total mineralization of the water (about 6 g L^–1^), estimated by summing the concentrations of ions, was only 17% of marine salinity. Therefore, most of the water is expected to be derived from meteoric recharge. The magnesium/strontium mass ratio was very low (0.06), suggesting that strontium accumulated in water during the prolonged residence time. The concentration of barium, a key indicator of sulfate removal by sufate reduction, was rather high (3.9 mg L^–1^). The concentration of sulfate was higher than in waters collected at the 3P Parabel (Frank et al., 2016) and 1-R Byelii Yar boreholes (Kadnikov et al., 2018) and varied from year to year (90.4 mg L^–1^ in 2016 and 23.3 mg L^–1^ in 2015). The water contained dissolved hydrogen sulfide at concentrations of 15.7 mg L^–1^ in 2016 and 7.6 mg L^–1^ in 2015.

**TABLE 1:** Physical and chemical characteristics of the water at 5P borehole.

**Parameter (unit)**	**Value**
Temperature (^*o*^C)	19.8
pH	7.5
Eh, mV	−329
Na (mg L^–1^)	1826
B (mg L^–1^)	4.7
K (mg L^–1^)	14.6
Ca (mg L^–1^)	233
Mg (mg L^–1^)	1.1
Sr (mg L^–1^)	17.0
Ba (mg L^–1^)	3.9
Si (mg L^–1^)	16.8
Fe (mg L^–1^)	1.5
Cl ^–^ (mg L^–1^)	3260
F ^–^ (mg L^–1^)	4.2
SO_4_^2–^ (mg L^–1^)	90.4
H_2_S (mg L^–1^)	15.7

The water flowing out from the borehole exsolved gas bubbles under atmospheric pressure. The gas contained mostly methane (81.1%) followed by nitrogen (16.3%) and oxygen (2.5%). The isotopic composition of methane (δ^13^C_*av*_. = −59.87–60.03‰) is consistent with its mostly biogenic origin (Kadnikov et al., 2017a).

### Metagenome Sequencing and Assembly of MAGs

In order to assemble the composite genomes of the most abundant members of the microbial community, we sequenced the metagenome of a water sample using the Illumina HiSeq2500 platform. A total of 16.9 Gbp metagenomic sequences were assembled into contigs, which were clustered into 136 genome bins. Seventy one bins representing MAGs with more than 85% completeness altogether comprised 93.3% of the whole metagenome (Table 2 and Supplementary Table S1). Analysis of the presence of a set of single-copy conserved genes revealed that 45 of these 71 bins could be classified as high-quality MAGs with more than 90% completeness and less than 5% contamination, as proposed by Bowers et al. (2017).

**TABLE 2:** General characteristics of high quality MAGs.

**MAG ID^*a*^**	**Completeness/contamination (%)**	**Contigs^*b*^**	**MAG size (Mbp)**	**Share in the metagenome (%)**	**Marker genes^*c*^**
***Euryarchaeaota***					
Ch96	100/0	3	1.83	2.65	*mcrA*
Ch26	99/0	5	1.56	2.47	*mcrA*
Ch131a	99/0	6	2.48	1.77	*mcrA*
Ch14	99/0	28	2.98	0.95	*mcrA*
Ch47	98/0	1	1.82	0.62	*mcrA*
***Armatimonadetes***					
Ch1	95/3	37	6.12	3.03	
Ch118	96/0	88	3.95	1.66	*cox, narG, norB, nrfA*
***Bacteroidetes***					
Ch61	98/1	100	3	1.42	*ntfA*
***Proteobacteria***					
Ch3	97/3	5	3.47	7.33	
Ch28	97/1	25	4.24	1.12	*dsrAB, nrfA*
Ch67	99/3	95	6.32	0.50	*cox, narG, nirS, norB, nosZ*
***Firmicutes***					
Ch130	97/3	55	2.71	5.51	
Ch29	96/1	36	3.35	4.20	
Ch97	97/1	39	2.58	2.28	
Ch121	95/2	22	3.37	1.81	
Ch109	99/4	55	4.77	1.04	
Ch98	99/3	165	3.15	0.77	
Ch38	98/0	49	3.13	0.63	
***Ignavibacteriae***					
Ch128a	94/1	1 (C)	2.4	12.08	*nrfA*
***Ca*. Kapabacteria**					
Ch6	96/1	1 (C)	2.66	1.28	*dsrAB, cox, nozZ, nrfA*
**CPR group**					
Ch104c	n/a	1 (C)	0.83	1.36	
Ch104b	n/a	1(C)	0.85	0.79	
***Thermotogae***					
Ch94	98/0	18	2.17	1.01	
**WOR-3**					
Ch92	96/0	5	1.96	1.05	
***Ca*. Riflebacteria**					
Ch46	98/2	35	5.39	0.83	

*^a^MAGs with > 90% completeness and < 5% contamination, with > 0.5% shares in the metagenome are listed. ^b^(C) indicates complete closed genome. ^c^The presence of the following genes is shown: mcrA (methyl-coenzyme M reductase subunit alpha), dsrAB (dissimilatory sulfate reductase subunits alpha and beta), cox (cytochrome c oxidase), narG (repiratory nitrate reductase subunit alpha), nirK/nirS (dissimilatory nitrite reductase nirK and/or nirS), norB (nitric oxide reductase subunit B), nosZ (nitrous-oxide reductase), and nrfA (ammonia-forming nitrite reductase, cytochrome c552 subunit).*

**FIGURE 1:**
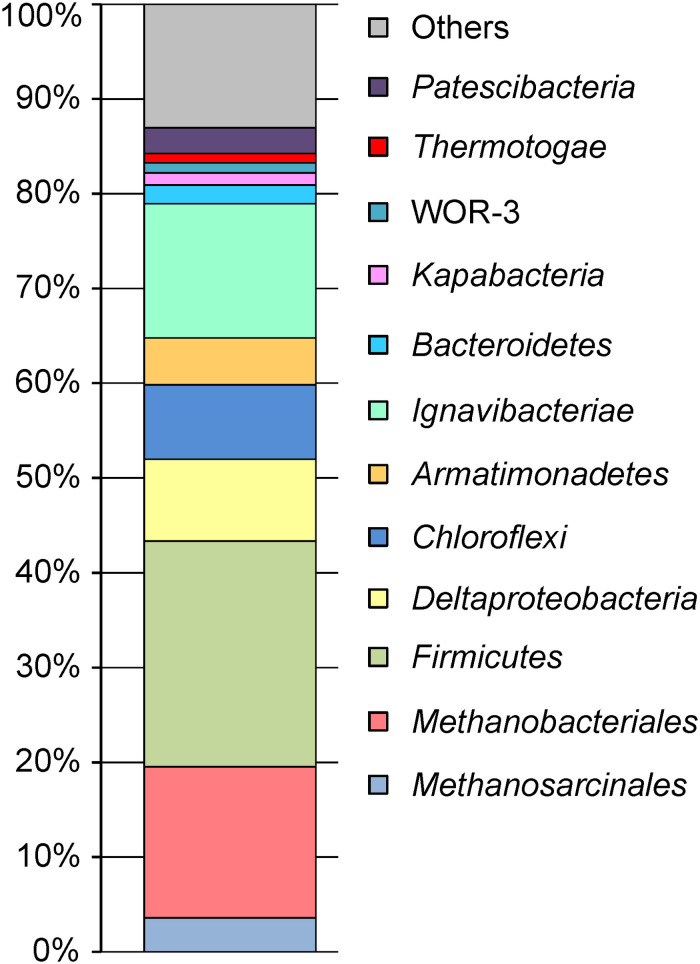
The relative abundance of taxonomic groups of microorganisms in the metagenome.

Phylogenetic identification of MAGs based on searches against the GTDB (Parks et al., 2018) revealed the same major prokaryotic lineages that were detected by 16S rRNA profiling (Kadnikov et al., 2017a): *Euryarchaeota* (20.4% of the whole metagenome), *Firmicutes* (24.1%), *Ignavibacteriae* (14.3%), *Proteobacteria* (9.2%), *Chloroflexi* (7.9%), *Armatimonadetes* (5.0%), *Bacteroidetes* (2.0%), *Thermotogae* (1.0%), the candidate phyla Kapabacteria (1.3%), and WOR-3 (1.1%) (Figure 1). Four MAGs assigned to the Candidate Phyla Radiation (CPR), a lineage comprising symbiotic or parasitic bacteria with a small cell size and reduced genomes (Castelle et al., 2018), accounted for about 2.8% of the metagenome. The actual relative abundance of CPR bacteria may be higher, because a 0.2-μm filter was used to collect the biomass, and small cells could pass through it. The shares of other identified phylum-level lineages, *Acidobacteria*, *Actinobacteria*, *Ca.* Aminicenantes, *Ca.* Atribacteria, *Ca.* Bipolaricaulota, *Coprothermobacterota, Nitrospirae, Ca.* Omnitrophica, *Ca.* Riflebacteria, *Spirochaetes*, and *Ca.* Verstraetearchaeota were less than 1% each (Supplementary Table S1).

### Methanogenic Archaea

All archaeal MAGs belonged to known methanogenic lineages, namely *Methanobacteriales* (Ch26, Ch35, Ch131a, and Ch131b), *Methanosarcinales* (*Methanosaetaceae*, Ch14 and Ch96), *Methanomassiliicoccales* (Ch47), *Ca.* Methanofastidiosales (Ch133), and *Ca.* Verstraetearchaeota (Ch88). Therefore, all three types of methanogens, hydrogenotrophic, acetoclastic and methyl-reducing, were present in the groundwater. Hydrogenotrophic methanogens predominated (16.1% of metagenome) and mostly belonged to the genus *Methanothermobacter* (Ch35 and Ch26 with average AAIs of 96.4 and 66.3% to *Methanothermobacter marburgensis* str. Marburg). According to the AAI thresholds proposed by Konstantinidis et al. (2017) for uncultivated microorganisms (45–65% for the same family, 65–95% for the same genus and 95–100% for the same species), Ch35 is a distinct strain of *M. marburgensis*, while Ch26 represented another species in this genus. The second most numerous group (3.6%) were acetoclastic methanogens of the family *Methanosaetaceae*, represented by *Methanosaeta thermophila* (Ch96–99.0% AAI to *Methanosaeta thermophila* PT) and a novel genus-level lineage (Ch14–61.7% AAI to *M. thermophila*). Another group of methanogens, members of the order of *Methanomassiliicoccales* that produces methane by reducing methanol and other methylated compounds with hydrogen as an electron donor (Lang et al., 2015), were less abundant (0.52%). *Methanomassiliicoccales* were represented by a single MAG Ch47 phylogenetically distant from known species of this order (47–59% AAI). Two other archaeal lineages present in minor amounts are likely characterized by a similar type of methanogenesis through reduction of methylated compounds. Ch133 belongs to the candidate genus Methanofastidiosum (81% AAI to Arc I group archaeon ADurb1213_Bin02801) of the euryarchaeal order *Ca.* Methanofastidiosales (Nobu et al., 2016) also known as WSA2 or Arc1. The second MAG, Ch88, belongs to the genus *Ca.* Methanosuratus (88.7% AAI to *Ca.* Methanosuratus petracarbonis) within the candidate phylum Verstraetearchaeota (Vanwonterghem et al., 2016). Notably, we found no organisms related to known anaerobic methane oxidizers (ANME-1, 2, 3).

### Firmicutes

The most abundant phylum, *Firmicutes*, accounted for 24.6% of the community and was represented by 21 MAGs. Only two of these MAGs could be assigned to known genera, while most others were phylogenetically distant from known lineages (Supplementary Table S1). Ch97 belongs to the genus *Pelotomaculum*, sharing 98.21% AAI with *P. thermopropionicum*, and Ch98 belongs to *Syntrophothermus*, sharing 95.7% AAI with *Syntrophothermus lipocalidus* DSM 12680. Members of these genera are mostly syntrophs growing in association with methanogens or anaerobic respiring organisms and capable of oxidizing propionate, fatty acids, alcohols or aromatic compounds (Sekiguchi et al., 2000; Imachi et al., 2002; Hidalgo-Ahumada et al., 2018).

The most abundant *Firmicutes* genotype, Ch130, accounting for 5.5% of the whole metagenome, may also have syntrophic metabolism. This MAG has an average AAI of 65.1% with *Thermacetogenium phaeum* DSM 12270, an anaerobic thermophilic bacterium oxidizing acetate in syntrophic association with a methanogenic partner (Hattori et al., 2000; Oehler et al., 2012). The presence of the Wood–Ljungdahl pathway used in the reverse direction for anaerobic acetate oxidation in *T. phaeum* in the Ch130 MAG indicates that this bacterium could have the same metabolism. *Firmicutes* phylogenetically related to *Thermacetogenium*, along with methanogenic archaea, were previously detected in another Western Siberian subsurface aquifer accessed via a 3P Parabel borehole (Frank et al., 2016).

Since sulfate-reducing members of the *Firmicutes* are common to the deep subsurface (e.g., Moser et al., 2005; Magnabosco et al., 2016; Jungbluth et al., 2017; Escudero et al., 2018) we searched for the presence of dissimilatory sulfate reduction pathways in all obtained *Firmicutes* MAGs. Surprisingly, only two MAGs, Ch2 and Ch87, whose shares in the genome were 1.61 and 0.24%, respectively, encoded the necessary set of enzymes and could be sulfate reducers. The presence of NrfAH ammonia-forming cytochrome *c* nitrite reductases in the MAGs Ch2 and Ch87 suggested that nitrite could also be used as an electron acceptor in anaerobic respiration. Both these MAGs are phylogenetically distant from cultured *Firmicutes* and belong to the candidate class DTU015 proposed in the GTDB taxonomy (Parks et al., 2018).

Ch19, assigned to the order *Limnochordales* (Watanabe et al., 2015) and accounting for about 0.37% of the metagenome, was the only *Firmicutes* MAG encoding cytochrome *c* oxidase, indicating the capacity for aerobic respiration. This bacterium also possesses a complete set of genes coding for dissimilatory nitrite reduction pathways that could operate under anaerobic conditions, including cytochrome *c* nitrite reductase NrfAH, NO-forming nitrite reductase NirK, nitric oxide reductase (Nor) and nitrous oxide (Nos) reductase. All other *Firmicutes* MAGs lacked known enzymes for dissimilatory reduction of oxygen, sulfate, nitrate and nitrite and are probably limited to anaerobic fermentative metabolism.

MAG Ch108, sharing 63.4% AAI with *Coprothermobacter proteolyticus* DSM 5265, could be assigned to the family *Coprothermobacteraceae*. *Coprothermobacter* species are proteolytic anaerobic fermentative bacteria (Etchebehere et al., 1998) phylogenetically distant from other *Firmicutes* and recently proposed to be considered as a distinct phylum *Coprothermobacterota* (Pavan et al., 2018).

### Chloroflexi

About 7.9% of the metagenome was assigned to the phylum *Chloroflexi*. All 12 *Chloroflexi* MAGs were assigned to the class *Anaerolinea*, members of which have been identified from diverse environments, including marine and freshwater sediments and deep subsurface aquifers (Yamada and Sekiguchi, 2009). They were also found in the Western Siberian deep subsurface (Kadnikov et al., 2018). Only one MAG, Ch72, was classified at the genus level, being assigned to the *Thermanaerothrix*, sharing 83.69% AAI with the only cultured species of this genus, thermophilic anaerobic bacterium *Thermanaerothrix daxensis*, isolated from a deep hot aquifer in France (Grégoire et al., 2011).

*Anaerolinea* are metabolically versatile heterotrophs capable of growing on various carbohydrates by fermentation, as well as aerobic and anaerobic respiration (Yamada et al., 2006). The search for respiratory pathways revealed the presence of cytochrome *c* oxidases in six MAGs, cytochrome *c* nitrite reductase NrfAH in four MAGs, NarGHI-type nitrate reductase in one MAG, NirK/NirS nitrite reductases in three MAGs and nitrous oxide (Nos) reductase in one MAG (Supplementary Table S1). Interestingly, contrary to *T. daxensis*, described as an anaerobic chemoorganotroph fermenting various carbohydrates, Ch72 contained cytochrome *c* oxidase and dissimilatory nitrite reductases and can probably obtain energy through respiration. Five *Chloroflexi* MAGs (Ch5, Ch9, Ch43, Ch80, and Ch93) do not contain any of the above-mentioned reductases. They also do not possess the complete set of genes of the TCA cycle and therefore are likely limited to a fermentative lifestyle.

### Deltaproteobacteria

*Deltaproteobacteria* accounted for 8.7% of the metagenome. The two most abundant genotypes, Ch3 (7.3%) and Ch 28 (1.1%), shared 54–55% AAI with *Syntrophus aciditrophicus* SB and were assigned to the family *Syntrophaceae* (recognized as the order *Syntrophales* in the GTDB taxonomy).

Cultured members of *Syntrophacea* are syntrophic organisms that degrade intermediates of organic decomposition, such as short-chain fatty and aromatic acids, to acetate, formate and hydrogen in co-culture with hydrogen-/formate-consuming methanogens or sulfate reducers (Elshahed et al., 2001; McInerney et al., 2007). Interestingly, these two MAGs have drastically different respiratory capacities. The more abundant organism, Ch3, appeared to lack known enzymes that enable dissimilatory reduction of sulfate, sulfite, nitrate and nitrite, as well as cytochrome *c* oxidases. Analysis of the Ch3 genome revealed the complete pathway for beta-oxidation of fatty acids, as well as enzymes involved in the utilization of butyrate and propionate. Hydrogen and formate could be formed as fermentation products, as evidenced by the presence of formate dehydrogenase and hydrogenase. The search for potentially secreted hydrolytic enzymes revealed the absence of signal peptide-containing glycosyl hydrolases and proteases. Probably, Ch3 bacterium is devoted to fermentation of low-molecular-weight fatty acids, presumably in syntrophic association with methanogenic archaea and/or sulfate reducers. The presence of multiple transporters for amino acids and peptides indicates the ability of Ch3 bacterium to ferment these substrates.

The genome of the second member of the *Deltaproteobacteria*, Ch28, was found to encode most of the enzymes of the dissimilatory sulfate reduction pathway, namely, sulfate adenylyltransferase, dissimilatory sulfite reductase (DsrAB and DsrC), sulfite reduction-associated complex DsrMKJOP, and the heterodisulfide reductase complex. However, genes for adenosine 5’-phosphosulfate reductase (AprAB) were not found. It is possible that Ch28 can reduce only sulfite rather than sulfate, or the *aprAB* genes were missed in the assembly. The presence of a [NiFe] group 1b hydrogenase that enables respiratory H_2_ uptake further supports the possibility of anaerobic respiration. Cytochrome *c* oxidases were not identified, but cytochrome *c* nitrite reductase NrfAH was encoded. Like the Ch3 bacterium, the Ch28 genome encodes the enzymes of the beta-oxidation pathway, indicating the possibility of utilization of fatty acids. The presence of an ABC transporter for the import of carbohydrates and amino acids/peptides likely indicates a broader substrate range of the Ch28 bacterium.

The third species, Ch74, assigned to the family *Syntrophobacteraceae*, represented a minor part of the community (0.26% of metagenome). Cultured members of this family are strictly anaerobic bacteria, having a respiratory or fermentative type of metabolism; some species form a syntrophic association with hydrogen/formate-utilizing partners (Kuever, 2014). *Syntrophobacteraceae* species are major acetate- and propionate-degrading sulfate reducers in paddy soil (Liu et al., 2018; Liu and Conrad, 2017). Analysis of the Ch74 genome revealed the *apr* and *dsr* genes of the sulfate reduction pathway, as well as *nrfAH* nitrite reductase and *norB* nitric oxide reductase.

### Armatimonadetes

The phylum *Armatimonadetes* was represented by three MAGs, and together they accounted for about 5% of the metagenome. Members of this bacterial phylum, initially found in Obsidian Pool, Yellowstone National Park and designated as OP10 (Hugenholtz et al., 1998), have been detected in numerous 16S rRNA gene surveys and metagenomic studies from various environments. A few cultured members of this phylum are aerobic heterotrophs growing on various sugars, including complex polysaccharides (Lee et al., 2011, 2014; Im et al., 2012; Li et al., 2019).

One MAG, Ch118, was assigned to the class *Chthonomonadetes*, comprising a single cultured member, *Chthonomonas calidirosea*, a thermophilic motile aerobic bacterium capable of utilizing a wide range of carbohydrates, including starch, carboxymethyl cellulose and xylan (Lee et al., 2011). Although Ch118 is phylogenetically rather distant from this species (47.5% AAI), its genome also encoded flagellar motility, an aerobic respiratory chain with cytochrome *c* oxidase and numerous carbohydrate-active enzymes and sugar transporters. Anaerobic respiration could be enabled by dissimilatory nitrate, nitrite, nitric-oxide, and tetrathionate reductases.

The most abundant *Armatimonadetes* MAG (3.3% of the metagenome), Ch1, was assigned to the candidate class UBA5377 in the GTDB taxonomy, still lacking cultured members. The third *Armatimonadetes* MAG, Ch33, belonged to the same class. Analysis of both these genomes revealed the absence of an aerobic respiratory chain, as well as pathways for dissimilatory reduction of sulfate and nitrate. Presumably, these two organisms are anaerobic fermenters, unlike all cultured *Armatimonadetes* species.

### *Ca.* Kapabacteria

The recently proposed candidate phylum Kapabacteria (Kantor et al., 2015), also known as the OPB56 clade within the Bacteroidetes/Chlorobi/Ignavibacteriae superphylum, accounted for 1.29% of the metagenome and comprised a single genotype, Ch6. The genome of this bacterium was assembled as a single circular 2,660,650 bp long chromosome; this is the first known complete genome of *Ca.* Kapabacteria. It was predicted to contain 2275 protein-coding genes, 47 tRNA genes and a single 16S-23S-5S rRNA operon.

Analysis of the Ch6 genome revealed the presence of a complete set of genes required for dissimilatory sulfate reduction, including sulfate adenyltransferase, adenisine-5’-phisphate reductase, dissimilatory sulfite reductase and related redox complexes Qmo and DsrMKJOP. These results are consistent with the finding of a sulfate reduction pathway in the partial genomes of another member of *Ca.* Kapabacteria, *Ca*. Thermonerobacter thiotrophicus, obtained from hot springs in United States and Japan (Thiel et al., 2019). Taking into account 96.81% AAI between Ch6 and *Ca*. T. thiotrophicus MAG Naka2016_bin-10 (GenBank GCA_003731655.1) from Japan, these two bacteria probably represent a single species. Like the latter genome, Ch6 MAG contained genes for NrfAH-type nitrite reductase, and nitrous oxide reductase (NosZ), cytochrome *bd* ubiquinol oxidase and an aerobic respiratory pathway with a terminal *aa3*-type cytochrome *c* oxidase. Additionally, the Ch6 genome encodes ArrAB-type respiratory arsenate reductase, absent in the Naka2016_bin-10 genome assembly. The presence of both *dsr* and *cox* genes is unusual since sulfate-reducing bacteria are unable to derive energy from aerobic respiration. Nevertheless, cytochrome *c* oxidase genes were found in *Desulfovibrio* genomes, and the ability of a mutant strain of *Desulfovibrio vulgaris* to grow with energy derived from oxidative phosphorylation linked to oxygen reduction was recently demonstrated (Schoeffler et al., 2019). Therefore, it is possible that the Ch6 bacterium is metabolically versatile and adapted to fluctuating concentrations of oxygen and alternative electron acceptors.

Analysis of the Ch6 genome revealed a very limited potential of this bacterium to utilize organic substrates. No presumably secreted carbohydrate-active and proteolytic enzymes were identified. We found no transporters for the import of sugars and only a few transporters of peptides and amino acids. A chemolithoautotrophic lifestyle is unlikely, since the genome lacked the Wood–Ljungdahl pathway typically used by sulfate reducers for carbon fixation, as well as other CO_2_ fixation pathways. Therefore, the Ch6 bacterium is probably a highly specialized organism with a narrow substrate range. The presence of acetyl-CoA synthetase (ADP-forming) suggests that the Ch6 bacterium could assimilate acetate and oxidize it in the course of aerobic or anaerobic respiration.

The Ch6 genome contained genes required for flagellar motility and chemotaxis, indicating that this bacterium is motile, like members of the *Bacteroidetes* and *Ignavibacteriae*. However, genes encoding the type IV pili for twitching motility were not found. The type IV pili enable attachment of the cells to insoluble substrates, and their absence is consistent with the predicted inability of Ch6 to hydrolyse polysaccharides.

### *Ignavibacteriae* Bacterium Ch128a

The phylum *Ignavibacteriae* was the second most numerous lineage in the microbial community of groundwater. It was represented by two MAGs, Ch128a, and Ch128b, accounting for 12.08 and 2.22% of the whole metagenome, respectively. Both MAGs were only distantly related to cultured species of this phylum, *Ignavibacterium album* (Iino et al., 2010) and *Melioribacter roseus* (Podosokorskaya et al., 2013).

The genome of the Ch128a bacterium was sequenced to 764x average coverage and assembled into a single circular chromosome with a length of 2,403,876 bp. Taking into account the abundance of this lineage, we took advantage of availability of a complete genome sequence for detailed analysis of the phylogenetic position and metabolic potential of the Ch128a bacterium.

As a result of genome annotation, 2,067 potential protein-coding genes were identified, and the functions of 1,017 (49%) of them were tentatively assigned. A single ribosomal RNA operon comprising the 16S-23S-5S rRNA genes and 44 transfer RNA (tRNA) genes were identified in the genome. The Ch128a cells were predicted to be rod-shaped, based on the identification of genes encoding the rod-shape determining proteins MreBCD, RodA and peptidoglycan D,D-transpeptidase MrdA. The bacterium is likely non-motile because it lacks flagellar machinery and chemotaxis genes.

**FIGURE 2:**
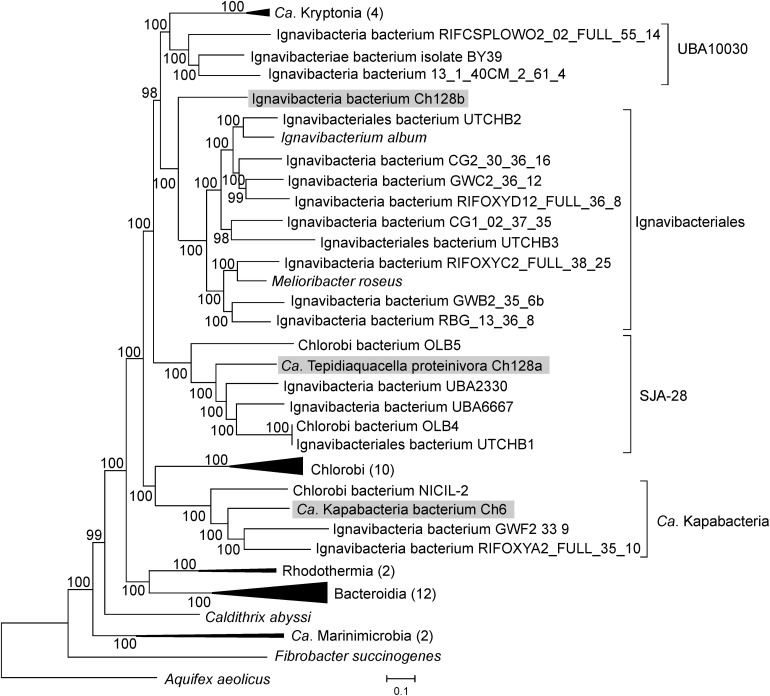
Phylogenomic placement of the Ch6, Ch128a, and Ch128b genomes in the maximum likelihood concatenated protein phylogeny of the Bacteroidetes/Chlorobi group. The level of support for internal branches was assessed using the Bayesian test in PhyML. The numbers of genomes used for the analysis in the collapsed clusters is shown after the name of the taxon. Taxonomy is shown according to the GTDB.

The Ch128a 16S rRNA gene shares only 86% sequence identity with the closest cultured bacterium, *Ignavibacterium album*. Several 16S rRNA sequences with about 90% identity (AY344403, RPQH01000040, QEVB01000012, MWSW01000018, etc.) were detected in freshwater lakes and bioreactor sludge. To determine the phylogenetic position of the Ch128a bacterium, a phylogenetic tree based on concatenated sequences of conservative marker genes of bacteria assigned to the Bacteroidetes/Chlorobi group (the phylum *Bacteroidota* in the GTDB taxonomy) was constructed. The results confirmed that Ch128a belongs to *Ignavibacteriae* and placed it within the candidate order SJA-28 (also known as Chlorobi lineage 4), as defined by the GTDB taxonomy (Figure 2). The AAI values between Ch128a and other SJA-28 genomes, OLB4, OLB5, UTCHB1, UBA2330 and UBA6667 was in the range of 48–53%. The taxonomic status of the SJA-28 lineage, as well as *Ignavibacteriae*, remained unclear. *Ignavibacteriae* have been described as a distinct phylum in the Bacteroidetes/Chlorobi group, along with *Bacteroidetes* and *Chlorobi* (Podosokorskaya et al., 2013), and such classification is currently used in the NCBI taxonomy database (Federhen, 2012). The GTDB taxonomy considers *Ignaviibacteria* as a class (comprising orders *Ignavibacteriales* and SJA-28) in the phylum *Bacteroidota*, along with the classes *Bacteroidia*, *Chlorobi*, *Rhodothermia*, *Ca.* Kapabacteria, UBA10030, and *Ca.* Kryptonia. Our phylogenetic analysis showed that SJA-28 is a distinct lineage within a clade comprising *Ignavibacteriales*, UBA10030 and *Ca.* Kryptonia (Figure 2). This whole group is separated from the *Chlorobi, Ca.* Kapabacteria and *Bacteroidetes/Rhodothermia* clades and thus could be considered as the phylum *Ignavibacteriae* or as the class *Ignaviibacteria* of the larger phylum *Bacteroidota.*

**FIGURE 3:**
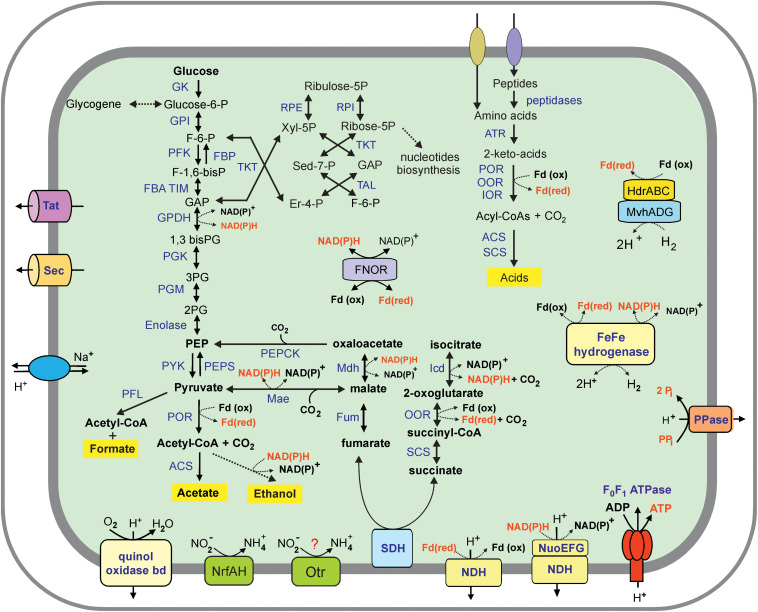
An overview of the metabolism of *Ignavibacteriae* bacterium Ch128a (*Candidatus* Tepidiaquacella proteinivora) reconstructed from its genome. Enzyme abbreviations: GK, glukokinase; GPI, glucose-6-phosphate isomerase; PFK, 6-phosphofructokinase; FBA, fructose-bisphosphate aldolase; TIM, triosephosphate isomerase; GPDH, glyceraldehyde 3-phosphate dehydrogenase; PGK, phosphoglycerate kinase; PGM, phosphoglycerate mutase; PYK, pyruvate kinase; PEPS, phosphoenolpyruvate synthase; FBP, fructose-1,6-bisphosphatase; PEPCK, phosphoenolpyruvate carboxykinase; RPE, ribulose-phosphate 3-epimerase; RPI, ribose 5-phosphate isomerase; TKT, transketolase, TAL, transaldolase, PFL, pyruvate formate lyase; POR, pyruvate ferredoxin oxidoreductase; ACS, acetyl-CoA synthetase, Mdh, malate dehydrogenase; Mae, malic enzyme; Fum, fumarate hydratase; SDH, succinate dehydrogenase; SCS, succinyl-CoA ligase; OOR, 2-oxoglutarate ferredoxin oxidoreductase; Icd, isocitrate dehydrogenase; ATR, aminotransferase; IOR, indolepyruvate ferredoxin oxidoreductase; FNOR, ferredoxin-NAD(P) + reductase; PPase, pyrophosphatase; NDH, NADH dehydrogenase (subunits NuoABCDHIJKLMN); Otr, octaheme tetrathionate reductase. Other abbreviations: F-6-P, fructose 6-phosphate; F-1,6-bisP, fructose-1,6-bisphosphate; GAP, glyceraldehyde-3 phosphate; 1,3–bis–PG, 1,3–biphosphoglycerate; 3–PG, 3–phosphoglycerate; 2–PG, 2–phosphoglycerate; PEP, phosphoenolpyruvate; Xyl-5P, xylulose 5-phosphate; Er-4-P, erythrose 4-phosphate; Sed–7-P, sedoheptulose–7–phosphate; Pi, phosphate; PPi, pyrophosphate.

Analysis of the Ch128a genome revealed that this microorganism has a limited capacity to degrade complex organic substrates. Contrary to cultured members of *Ignavibacteriae*, *M. roseus* and *I. album*, metabolically versatile facultative anaerobes growing on various carbohydrates (Liu et al., 2012; Kadnikov et al., 2013), the Ch128a bacterium lacked secreted carbohydrate-active enzymes and thus was predicted to be unable to utilize polysaccharides. A few encoded glycoside hydrolases lacked N-terminal secretion signals and are likely involved in the synthesis and degradation of a storage polysaccharide, glycogen. Consistently, the Ch128a genome encodes a complete set of genes for the Embden-Meyerhof pathway of glycolysis and gluconeogenesis, as well as the non-oxidative branch of the pentose phosphate pathway (Figure 3). The ability of the Ch128a bacterium to utilize proteinaceous substrates is indicated by the presence of five signal-peptide-containing proteases belonging to the M3, M13, M16, M28, and C1 families, and amino acid transport systems. The imported amino acids can then be deaminated by a number of aminotransferases in glutamate dehydrogenase coupled reactions, followed by oxidation to generate the corresponding coenzyme A (CoA) derivatives (Figure 3). This step could be performed by ferredoxin-dependent oxidoreductases with different substrate specificities, including pyruvate: ferredoxin oxidoreductase, indolepyruvate: ferredoxin oxidoreductase and 2-oxoglutarate ferredoxin oxidoreductase. Pyruvate could also be used by pyruvate formate lyase, which catalyses the conversion of pyruvate and CoA to formate and acetyl-CoA. The acyl-CoA derivatives in Ch128a could be oxidized to the corresponding acids by acetyl-CoA synthetase and succinyl-CoA synthetase with concomitant generation of ATP. The presence of aldehyde and alcohol dehydrogenases suggested that alcohols could be produced as fermentation products. NADH and reduced ferredoxin, generated in fermentation pathways, could be re-oxidized by cytoplasmic [FeFe] group A hydrogenase and group 3c [NiFe] heterodisulfide reductase-linked hydrogenase (MvhADG/HdrABC) that bifurcates electrons from H_2_ to heterodisulfide and ferredoxin (Kaster et al., 2011).

The key enzymes of autotrophic carbon fixation pathways, the Calvin-Benson-Bassham, 3-hydroxypropionate and Wood–Ljungdahl pathways were not found. The tricarboxylic acid cycle is incomplete due to the lack of citrate synthase and aconitate hydratase and is likely used only for biosynthetic purposes.

Analysis of the Ch128a genome reveal the presence of subunits of the proton-translocating NADH-dehydrogenase complex, while the cytochrome *bc*_1_ complex, quinol-oxidizing alternative complex III, and terminal cytochrome *c* oxidases were not found, suggesting the inability of the Ch128a bacterium to grow by aerobic respiration. The only terminal reductase, the quinol oxidase *bd* complex, is probably involved in oxygen detoxification rather than respiration, as in most anaerobic bacteria. The Ch128a genome contained several clusters and separate genes encoding a full set of 14 NADH dehydrogenase subunits: *ndhABCDHIJKLMN*, *ndhABCDEF*, *ndhKL*, *ndhG*, *ndhH*, *ndhJ*, *ndhM*, and *ndhAB*. However, the absence of *nuoEFG* genes encoding the NADH-interacting module (Sazanov, 2007) in the first cluster suggests that it could encode an alternative proton-transporting complex that accepts electrons from reduced ferredoxin rather than NADH, as suggested for *M. roseus* (Kadnikov et al., 2013). The generated transmembrane ion gradient can be used for ATP generation by membrane F_0_F_1_-type ATP synthase (Figure 3).

Genome analysis revealed only two potential terminal reductases of anaerobic respiratory pathways. The first one is an ammonia-forming cytochrome *c* nitrite reductase of the NrfAH-type. The second gene cluster encodes the octaheme tetrathionate reductase (Otr) and cytochrome *b561* protein. The Otr enzymes are responsible for the reduction of tetrathionate to thiosulfate but could also be involved in nitrite reduction (Simon et al., 2011). Both predicted reductases contain subunits with transmembrane helices and are probably linked to the cytoplasmic membrane where they could accept electrons from the quinone pool. The presence of this enzyme can enable the use of nitrite and sulfur compounds under anaerobic conditions. The genome of Ch128a is the first complete genome of a member of the SJA-28 lineage of the phylum *Ignavibacteriae*, and we propose the following taxonomic names for the novel genus and species of Ch128a:

**Description of *Candidatus* Tepidiaquacella gen. nov.**

*Candidatus* Tepidiaquacella (Te.pi.di.a.qua.cel’la, L. adj. *tepidus* warm; L. n. *aqua*, water; L. fem. n. *cella*, cell; N.L. fem. n. *Tepidiaquacella*, a cell from warm water).

**Description of *Candidatus* Tepidiaquacella proteinivora sp. nov.**

*Tepidiaquacella proteinivora* (pro.te.i.ni’vo.ra N.L. neut. n. *proteinum* protein; L. fem. suff. *vora* devouring; N.L. fem. adj. *proteinivora* devouring proteins).

Not cultivated. Inferred to be a rod-shaped, non-motile, anaerobic, obligate organotroph. Obtains energy by fermentation or respiration with nitrite and is able to use proteinaceous substrates for growth. Represented by the complete genome (GenBank CP054675) obtained from metagenome of a deep subsurface thermal aquifer in Western Siberia, Russia.

On this basis, we propose the following names for the order and family:

*Candidatus* Tepidiaquacellales ord. nov.

*Candidatus* Tepidiaquacellaceae fam. nov.

Assuming the results of phylogenetic analysis, and because of the appearance of a representative with known complete genome sequence, the SJA-28 lineage is proposed to be named as *Candidatus* Tepidiaquacellales ord. nov. within the class *Ignavibacteria* in the phylum *Ignavibacteriae* (Podosokorskaya et al., 2013). The order *Candidatus* Tepidiaquacellales is defined on a phylogenetic basis by comparative genome sequence analysis of *Candidatus* Tepidiaquacella proteinivora Ch128a, OLB4, OLB5, UTCHB1, UBA2330, and UBA6667.

### Diversity of Environmental Conditions and Microbial Processes in the Deep Subsurface Aquifer

Metagenomic analysis of the underground water reservoir revealed a diverse microbial community. First, it should be emphasized that the analyzed aquifer is located in Mesozoic sedimentary rocks, formed from sediments of marine origin. It is these deposits that became the basis for the formation of oil and gas fields in Western Siberia. This buried and completely or partly altered organic matter can support the development of organotrophic microbial communities that differ from the lithoautotrophic communities of oligotrophic subsurface ecosystems in which molecular hydrogen of abiotic origin is the main source of energy (Takai et al., 2004; Chivian et al., 2008; Pedersen, 2012).

**FIGURE 4:**
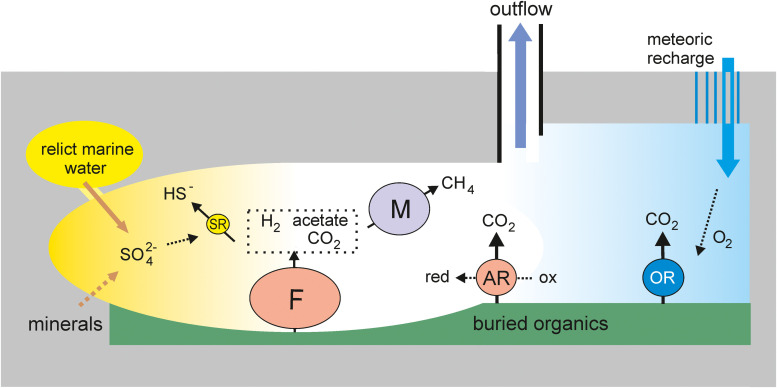
An ecological model of the deep subsurface aquifer. OR, aerobic respiration; F, fermentation; SR, sulfate reduction; AR, anaerobic respiration (other than SR); M, methanogenesis. The sizes of the circles roughly reflect the relative abundance of the respective metabolic groups in the metagenome.

Metagenomic analysis revealed four main metabolic groups of microorganisms (Figure 4). The first group is methanogenic archaea, which accounted for about 20% of the community. The most numerous among them were hydrogenotrophic methanogens of the order *Methanobacteriales*, which are widespread in various subsurface ecosystems, including deep underground aquifers in Western Siberia (Frank et al., 2016). The presence of acetoclastic and methyl-reducing methanogens indicates the use of not only hydrogen (which can be of both biogenic and abiogenic origin), but also products of microbial metabolism for methanogenesis. The second important functional group is sulfate reducers. Like methanogens, they are typical members of both lithoautotrophic and organotrophic microbial communities of the deep subsurface ecosystems, including Western Siberia (Moser et al., 2005; Chivian et al., 2008; Frank et al., 2016; Kadnikov et al., 2018). The sulfate reduction pathway was revealed in two genomes of *Firmicutes* (Ch2 and Ch87) and in the members of *Deltaproteobacteria* (Ch28 and Ch74), *Ca.* Kapabacteria (Ch6), and *Nitrospirae* (*Thermodesulfovibrio aggregans*, Ch24). In total, sulfate reducers accounted for only about 5% of the community, which is a much smaller proportion than in the microbial communities of aquifers accessible through boreholes 3P Parabel (∼50%) and 1-R Byelii Yar (∼30%). Considering that the concentration of sulfate in 5P water is several times higher than at these two boreholes (<5 mg L^–1^, Frank et al., 2016; Kadnikov et al., 2018), a lower relative abundance of sulfate reducers is unexpected, but this may be explained by a higher total concentration of microorganisms in the water collected at the 5P borehole. Alternatively, a higher concentration of sulfate in 5P water could be a consequence of lower abundance of sulfate-reducers and an overall low rate of the sulfur cycle.

The third major functional group is organotrophs with aerobic respiratory pathways, marked by the presence of cytochrome *c* oxidase. These include MAGs from the phyla *Chloroflexi* (Ch21, Ch27, Ch132, Ch71, Ch72, and Ch58), *Firmicutes* (Ch19), *Ignavibacteriae* (Ch128b), *Gammaproteobacteria* (Ch67), *Armatimonadetes* (Ch118), *Bacteroidetes* (Ch62), *Ca.* Omnitrophica (Ch114), and *Acidobacteria* (Ch122). The cytochrome *c* oxidase genes are also present in sulfate reducers assigned to *Ca.* Kapabacteria (Ch6). Some of these genomes also contain genes for enzymes involved in the oxidation of sulfur compounds. In total, organotrophs potentially capable of aerobic respiration accounted for 13% of the community.

Most of the community is composed of anaerobic, presumably heterotrophic microorganisms with a fermentative type of metabolism; the genomes of many of them also contain enzymes for the dissimilatory reduction of nitrite, nitrate, Fe (III), thiosulfate, tetrathionate, polysulfides, etc. This metabolic group includes most *Firmicutes*, some *Chloroflexi*, the most abundant lineages of *Ignavibacteriae* (Ch128a), *Deltaproteobacteria* (Ch3), *Armatimonadetes* (Ch1 and Ch33), and *Bacteroidetes* (Ch61), as well as a number of less abundant phyla (WOR-3, *Thermotogae, Ca.* Riflebacteria, *Spirochaetes, Ca.* Bipolaricaulota, *Actinobacteria, Ca. Aminicenantes*, and *Ca.* Atribacteria). The genomes of some anaerobic members of *Firmicutes* and *Chloroflexi* encoded a Wood–Ljungdahl pathway that can be used for autotrophic carbon fixation. These microorganisms can produce acetate and hydrogen consumed by sulfate reducers and methanogens. Fermentative *Firmicutes* and *Deltaproteobacteria* capable of oxidizing low-molecular-weight organics could be involved in syntrophic associations with methanogens or anaerobic respiring organisms. An important role for syntropy in deep subsurface environments has been reported (Gray et al., 2010; Kimura et al., 2010; Matsushita et al., 2020).

Some of the functional groups of microorganisms described above, for example, methanogens and aerobic heterotrophs, have mutually exclusive requirements regarding environmental conditions, which indicates the heterogeneity of conditions in the deep subsurface aquifer (Figure 4). The capacity for aerobic respiration is a rather unexpected property for microorganisms of the deep underground biosphere, which is considered as a strictly anaerobic habitat (Teske, 2005). However, their presence is consistent with the presence of several percent oxygen in dissolved gas. Oxygen was also found in gas samples taken over several years from 3P Parabel and 1-R Byelii Yar boreholes (Frank et al., 2016; Kadnikov et al., 2018). It can be proposed that oxygen and aerobic microorganisms were delivered to the reservoir from the surface, with meteoric recharge waters. Favorable conditions for the development of aerobes can be formed locally in the zones of entry of such waters into the aquifer, and sulfide oxidation with the formation of sulfate can also occur in them. In other parts of the underground aquifer system, strictly anaerobic conditions favorable to fermentative microorganisms, as well as sulfate reducers and methanogens consuming their metabolic products, can be maintained. Such respiratory versatile bacteria as Ch6 Kapabacteria, predicted to be able to use both oxygen and sulfate for respiration, could grow successfully at the oxic-anoxic interface. Sulfate reducers and methanogens probably develop in spatially separated parts of the aquifer, respectively, those rich and poor in sulfate. Sulfate could originate from the oxidation of sulfide minerals or the dissolution of sulfates, such as gypsum (calcium sulfate). The sulfate moiety from sedimentary low-soluble sulfates, such as barite (barium sulfate) and celectine (strontium sulfate), can also be used by sulfate reducing bacteria as an electron acceptor (Karnachuk et al., 2002). Sulfate could also remain in relict waters of marine origin. The observed temporary fluctuations in the sulfate content in the water collected at the borehole may reflect the flow of water from different parts of the reservoir.

It should be mentioned that the above four functional groups of prokaryotes were also detected in two other previously characterized deep subsurface aquifers in Western Siberia, albeit in various combinations. Methanogens, sulfate reducers and a small fraction of anaerobic heterotrophs were found in the 3P Parabel borehole, while potential aerobes were missing (Frank et al., 2016). Sulfate reducers, as well as aerobic and anaerobic heterotrophs, were found in the 1-R Byelii Yar borehole, but methanogens were nearly absent (Kadnikov et al., 2018). Apparently, the composition of the aquifer community as a whole is determined by the rate of meteoric recharge, which delivers oxygen, and the availability of sulfate. Thus, the water from the 3P Parabel borehole had the highest salinity (13–14 g L^–1^), which indicates the minimal inflow of oxygenated water from the surface, and a predominantly anaerobic chemolithoautotrophic community of sulfate reducers and methanogens became formed.

Overall, the availability of buried organic matter of marine sediments of the Mesozoic era and the spatial heterogeneity of the underground aquifer create the conditions for the development of microbial communities, taxonomically and functionally much more diverse than those found in oligotrophic underground ecosystems.

## Data Availability Statement

The datasets presented in this study can be found in online repositories. The names of the repository/repositories and accession number(s) can be found at: https://www.ncbi.nlm.nih.gov/genbank/, SRR6186653; https://www.ncbi.nlm.nih.gov/genbank/, SRR11854357; and https://www.ncbi.nlm.nih.gov/genbank/, PRJNA414521.

## Author Contributions

NR designed the research project and drafted the manuscript. VK and AM performed metegenome sequencing. VK, AB, OK, and NR analyzed the data. All authors read and approved the manuscript.

## Conflict of Interest

The authors declare that the research was conducted in the absence of any commercial or financial relationships that could be construed as a potential conflict of interest.

## Footnotes

1 https://github.com/najoshi/sickle

2 http://www.cbs.dtu.dk/services/SignalP/

3 http://www.compgen.org/tools/PRED-TAT/

4 http://www.cbs.dtu.dk/services/TMHMM/
